# Sequential Cytokine-Induced Killer Cell Immunotherapy Enhances the Efficacy of the Gemcitabine Plus Cisplatin Chemotherapy Regimen for Metastatic Nasopharyngeal Carcinoma

**DOI:** 10.1371/journal.pone.0130620

**Published:** 2015-06-22

**Authors:** Yin Li, Ke Pan, Li-zhi Liu, Yong-qiang Li, Mo-fa Gu, Hua Zhang, Wei-xi Shen, Jian-chuan Xia, Jian-jun Li

**Affiliations:** 1 State Key Laboratory of Oncology in South China; Collaborative Innovation Center for Cancer Medicine, Sun Yat-sen University Cancer Center, Guangzhou, 510060, P. R. China; 2 Department of Endoscopy, Sun Yat-sen University Cancer Center, Guangzhou, 510060, P. R. China; 3 Department of Biotherapy, Sun Yat-sen University Cancer Center, Guangzhou, 510060, P. R. China; 4 Department of Imaging and Invention Radiology, Sun Yat-sen University Cancer Center, Guangzhou, 510060, P. R. China; 5 Department of Radiation, Sun Yat-sen University Cancer Center, Guangzhou, 510060, P. R. China; 6 Cancer Center, The People’s Hospital of Guangdong Province, Guangzhou, 510089, P. R. China; 7 Cancer Institute, The Second Clinical Medical College, Jinan University, Shenzhen People's Hospital, Shenzhen, China, Shenzhen, 518020, P. R. China; Gustave Roussy, FRANCE

## Abstract

In this study, we investigated the efficacy of sequential cytokine-induced killer cell (CIK) immunotherapy with gemcitabine plus cisplatin (GC) regimen chemotherapy in metastatic nasopharyngeal carcinoma (NPC) patients. Between September 2006 and April 2010, 222 NPC patients with distant metastasis after radiotherapy completion were retrospectively analyzed: 112 patients received 4–6 cycles of GC chemotherapy at 4-week intervals, followed by at least 4 cycles of CIK immunotherapy at 2-week intervals (GC+CIK group); the remaining 110 patients received 4–6 cycles of GC chemotherapy alone (GC group). The evaluation of long-term efficacy showed that the progression-free survival (PFS) rate was significantly higher in the GC+CIK group (log-rank test; p = 0.009), as was the overall survival (OS) rate (p = 0.006). In conclusion, sequential CIK treatment may be effective in enhancing the therapeutic efficacy of GC chemotherapy for metastatic NPC patients. This study provides a basis for alternative therapeutic strategies for metastatic NPC.

## Introduction

Nasopharyngeal carcinoma (NPC) is one of the leading malignant tumors endemic in Southern China and Southeast Asia [1]. NPC has metastatic potential. Distant metastasis and recurrence have often been reported in patients who undergo radiotherapy. The liver, lung, and bone are the main sites of distant metastasis [2], and patients with distant metastasis usually have poor prognosis [3]. In recent years, a variety of comprehensive therapies based on chemotherapy as a palliative treatment for advanced NPC patients have been reported [4–6]. At Sun Yat-sen University Cancer Center, we first used gemcitabine (GEM) plus cisplatin (DDP) regimen (GC chemotherapy) in clinic practice as a first-line neoadjuvant chemotherapy regimen for patients with locoregionally advanced NPC. This chemotherapy regimen showed a higher response rate and better long-term efficacy than other traditional regimen chemotherapies [7]. However, for patients with distant metastasis after radiotherapy, more effective therapy methods still need to be investigated.

Transfusion of cytokine-induced killer cells (CIKs) is one type of adoptive cell therapy (ACT). CIKs are a population of heterogeneous cells generated in vitro by amplifying peripheral blood mononuclear cells (PBMCs) with multiple cytokines including IFN-γ, IL-2 and anti-CD3 monoclonal antibody [8]. CIK cells co-express the T cell marker CD3 and NK cell marker CD56, which can kill a broad range of tumor cells both in vitro and in vivo via non-MHC restriction [9,10]. Transfusion of CIK cells has been used as palliate or adjuvant treatment for solid tumors, such as hepatocellular carcinoma, gastric cancer, lung cancer and renal cell carcinoma, and some patients have achieved promising outcomes [11–16].

Previously, we investigated the efficacy of GC chemotherapy combined with autologous CIK infusion for NPC patients with distant metastasis after radiotherapy [17]. However, those results were limited by small sample size. Subsequently, in the present study, we further investigated the efficacy of GC chemotherapy with subsequent CIK immunotherapy for metastatic NPC patients in a relatively larger sample size of 222 cases. Our data provide additional evidence on the clinical effectiveness of GC chemotherapy plus CIK immunotherapy for metastatic NPC patients.

## Patients and Methods

### Patient selection

Between September 2006 and April 2010, a total of 306 NPC patients with distant metastasis after radiotherapy from three medical institutes in southern China (Sun Yat-sen University Cancer Center, Cancer Center at The People’s Hospital of Guangdong Province, and Cancer Institute at The People’s Hospital of Shenzhen City) were included in this retrospective analysis. All of the patients met the following criteria: (1) had undifferentiated, non-keratinizing carcinoma at the initial diagnosis (WHO, 1991 criteria) and no evidence of distant metastasis identified before radiotherapy [18]; (2) in coordination with radiotherapy, received regular chemotherapy with cisplatin, carboplatin, 5-flurouracil, paclitaxel, or other cytotoxic agent at standard doses, approximately 50–70 Gy, in the nasopharynx and neck. Within 3 months after radiotherapy, no local and distant lesions were found; (3) during regular follow-up, the distant metastatic lesions were detected by imaging more than 6 months after radiotherapy was completed; (4) did not receive chemotherapy or immunotherapy during the time between the completion of radiotherapy and the confirmation of the distant metastatic lesion.

### Study protocol

**Fig 1:**
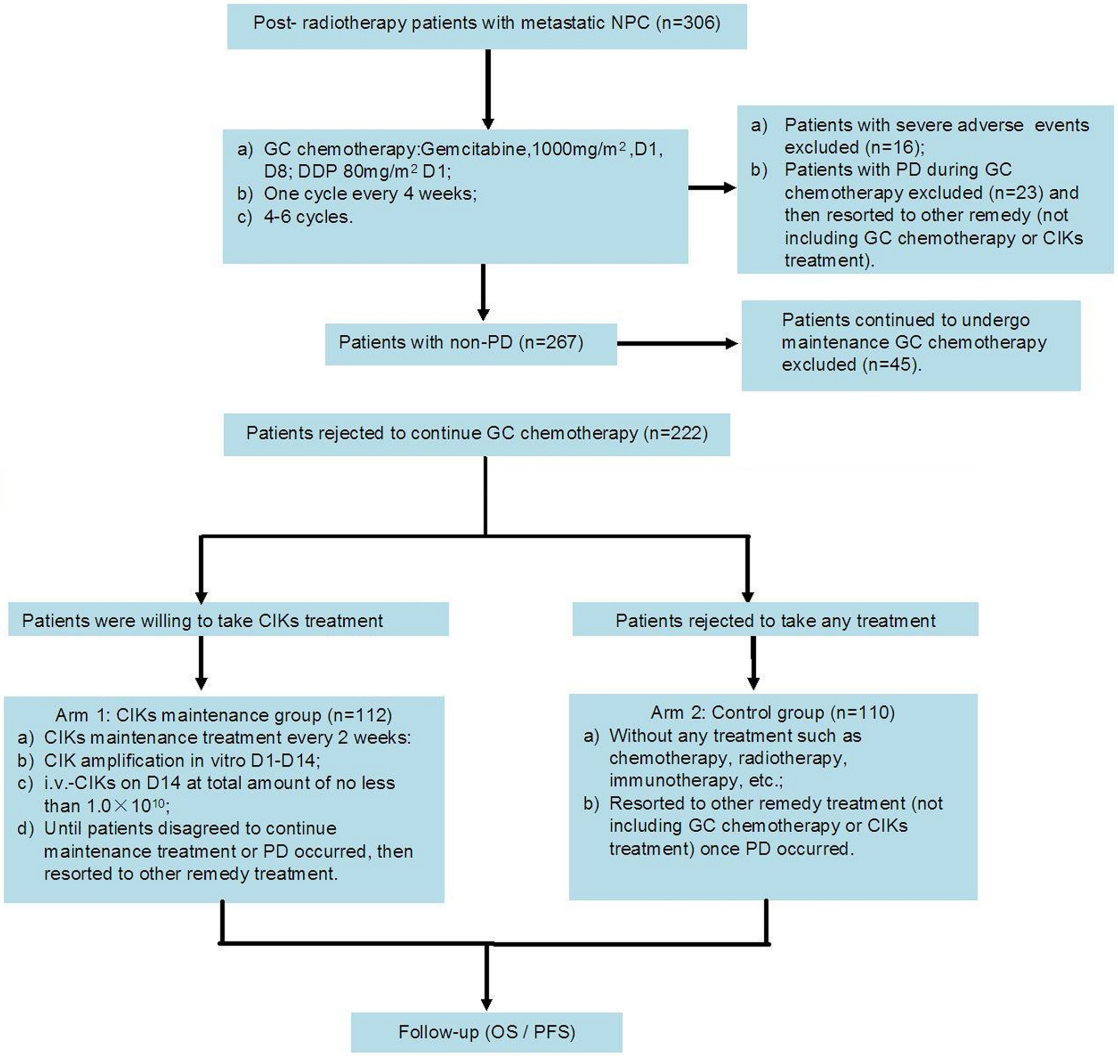
Study flow diagram.

The study protocol was approved by the ethics committees of Sun Yat-sen University Cancer Center, The People’s Hospital of Guangdong Province and The People’s Hospital of Shenzhen. Treatments were conducted in accordance with the approved guidelines. All NPC patients received 4–6 cycles of GC chemotherapy. Patients with severe adverse events (n = 16) and progressive disease (PD) (n = 23) during GC chemotherapy were excluded, as undergoing GC chemotherapy was deemed to be inappropriate for them. Also excluded were 45 patients who received greater than 4–6 cycles of maintenance GC chemotherapy. The remaining 222 patients were included in further analysis. The GC+CIK group/Arm 1, consisted of 112 cases who received sequential CIK maintenance treatment (at least 4 cycles of autologous CIK transfusion in 2-week intervals). The control GC group/Arm 2 included 110 patients who refused any treatment including chemotherapy, immunotherapy, and radiotherapy. If PD was detected in patients in either group during follow up, patients then resorted to other treatment options that did not include GC chemotherapy or CIK infusion. The uniform study protocol based on GC chemotherapy and sequential CIK immunotherapy was approved by the respective institutional review boards of the three medical institutes. All of the patients provided their signed informed consent before receiving GC chemotherapy or CIK treatment. The flow diagram of this study is shown in Fig 1.

### GC chemotherapy regimen

GEM (Zefei, Jiangsu Hansoh Pharmaceutical Co. Ltd., China) was administered at a dose of 1000 mg/m^2^ in 100 ml of normal saline by intravenous infusion for 30 min on D1 and D8. DDP (Nuoxin, Jiangsu Hansoh Pharmaceutical Co. Ltd., China) was administered at a dose of 20 mg/m^2^ in 500 ml of normal saline by intravenous infusion from D1 to D5. This treatment was administered every 4 weeks for 4–6 cycles.

### Preparation of autologous CIK cells and therapy procedure

**Fig 2:**
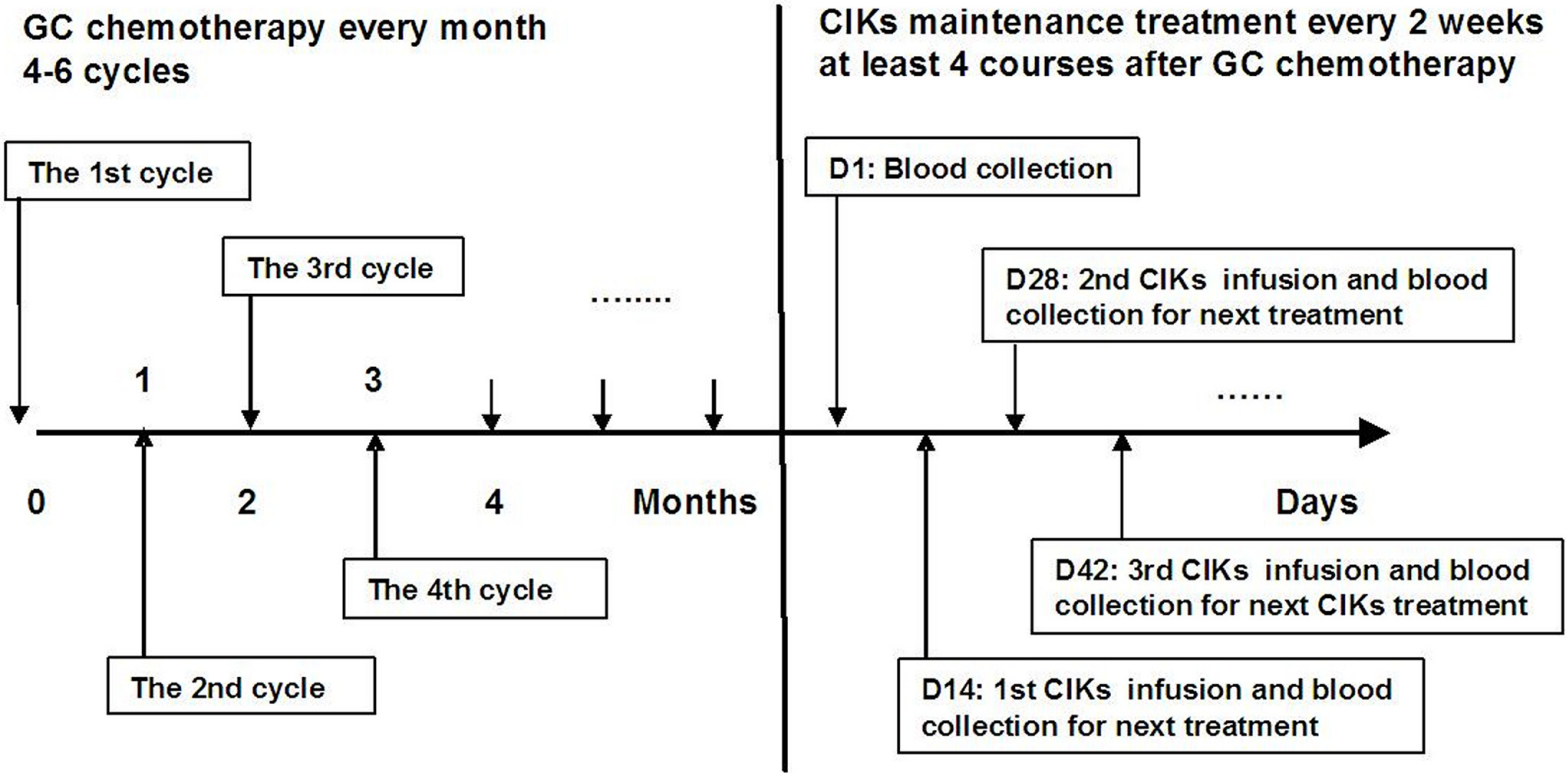
Schematic diagram of GC chemotherapy plus CIK treatment in metastatic NPC patients. All patients underwent 4–6 cycles of gemcitabine plus cisplatin regimen chemotherapy (GC chemotherapy) at 4-week intervals; over 2weeks later, a group of 112 patients received sequential treatment with autologous cytokine-induced killer cells (CIKs) for at least 4 cycles at 2-week intervals.

CIK cells were prepared as previously described [11,12,17]. Briefly, more than 2 weeks after the last GC chemotherapy, 50 ml of heparinized peripheral blood was collected from patients. Mononuclear cells were isolated by Ficoll density-gradient centrifugation and cultured using complete medium containing 1000 U/mL IFN-γ (Clone-gamma, Shanghai Clone Company, Shanghai, China) for 24 h. Mouse anti-human CD3 monoclonal antibody (R&D Systems, Shanghai, China), IL-2 (rhIL-2; Beijing Sihuan, Beijing, China) and IL-1α (Life Technologies, Guangzhou, China) were then added to a final concentration of 100 ng/ml, 1000 U/ml and 100 U/ml, respectively. Every 2–3 days, half of the medium was replaced with fresh complete medium containing 1000 U/ml IL-2 and cell density was maintained at 2 ×10^6^ cells/ml. After approximately 14 days of culturing, autologous CIK cells were harvested. Prior to administration, the CIK cells were assessed for viability by the dye exclusion test and checked twice for possible contamination by bacteria, fungi, and endotoxin. For administration, harvested CIK cells were washed and re-suspended with 100 ml of normal saline containing 3–5 ml of 20% human serum albumin. The autologous CIK cells were then administered via intravenous infusion over 30 min. During transfusion, vital signs such as pulse, heart rate, breathing rate, blood pressure and temperature were monitored and recorded. Maintenance CIK treatment was given every 2 weeks with at least 4 cycles performed in each patient. The treatment procedure is shown in Fig 2. If cases were classified by medical imaging as complete remission (CR), partial remission (PR) or stable disease (SD), the maintenance CIK treatment continued. However, if PD was detected or patients refused to continue participation, CIK treatment was discontinued and an alternative therapy was recommended by physicians.

### Toxicity associated with chemotherapy and CIK immunotherapy

The toxicity of chemotherapy was graded by the National Cancer Institute (1999) classification criteria [19]. The toxicity of CIK immunotherapy was evaluated according to our previous study [17].

### Evaluation of therapeutic efficacy by imaging

At baseline and every 2 months after the initiation of treatment with GC chemotherapy alone or in combination with CIK immunotherapy, metastasis in the liver, lung or distant lymph nodes was evaluated using computed tomography (CT) or magnetic resonance imaging (MRI) scan. Bone metastases were assessed by emission computed tomography (ECT) or MRI. The treatment efficacies were determined by the same imaging expert. According to the response evaluation criteria in solid tumors (RECIST, 1.0), patients were classified as having CR, PR, SD, or PD [20].

### Follow-up

Patient follow-up began the first time distant NPC metastasis was detected after the initial radiotherapy. All patients were followed every two months at our outpatient clinic. At each follow-up visit, pulmonary and abdominal CT, nasopharyngeal and cervical MRI as well as ECT scans of whole-body bone were performed. The deadline to schedule a follow-up visit was May 31, 2013. Progression-free survival (PFS) was calculated as the period from the date of the initial GC chemotherapy to the time at which visible tumor progression was first observed by imaging. Overall survival (OS) was calculated as the period from the date of the initial GC chemotherapy to the time of death or the last follow-up visit. As previously stated, once patients presented with PD at the follow-up visit, the GC chemotherapy or CIK treatment was discontinued and alternative treatment was recommended.

### Statistical analysis

The survival curves for OS and PFS were plotted using the Kaplan-Meier method, and the 1-year, 2-year and 3-year survival rates were determined by a life table. The log-rank test was used to compare the survival rates of patients between the two groups. For comparison of clinical parameters and short-term treatment efficacy between the two groups, the t-test, χ^2^ test, and Fisher exact test were used as appropriate. SPSS17.0 was used for statistical analysis, and p < 0.05 indicated statistical significance.

## Results

### Patient characteristics

In this study, 222 NPC patients divided into two arms, the GC+CIK group and GC group, were analyzed. There was no significant difference with regard to age, gender, ECOG score, association with nasopharyngeal recurrence lesions, organ distribution of metastasis and previous therapy. The clinical characteristics of the two patient groups are shown in Table 1.

**Table 1:** Baseline characteristics of NPC patients with distant metastasis (n = 222).

Patient characteristics	Am1:GC+CIK group (n = 112)	Am2:GC group (n = 110)	Chi-square value	p value
Sex			0.3021	0.5826
male	83	85		
female	29	25		
Age (years)				
mean	44.6	45.3		
range	32–63	33–62		
Performance status (ECOG)			0.1577	0.6913
0	52	54		
1	60	56		
Accompanying loco-regional disease#			0.8018	0.3705
Yes	12	8		
No	100	102		
Distant disease site			4.575	0.3338
Liver only	34	38		
Lung only	22	24		
Bone only	24	16		
Lymph node*	10	12		
Multiple sites	22	12		
No. of previous chemotherapy cycles			0.7035	0.8724
0	18	21		
1	21	20		
2	23	25		
≥3	50	44		
Previous chemotherapy agent			0.3711	0.9461
Cisplatin only	48	46		
5-flurouracil +cispalatin (PF)	26	25		
Paclitaxel + carboplatin (TC)	20	23		
Others	18	16		
Previous chemotherapy regimen			4.690	0.1960
Neoadjuvant chemotherapy	27	19		
Adjuvant chemotherapy	30	22		
Concomitant chemo-radiotherapy	40	54		
Others	15	15		
No. of CIK infusion cycles			/	/
≤ 8	31	/		
9–12	49	/		
> 12	32	/		

# Loco-regional disease included recurrent lesions in the nasopharynx or cervical /retropharyngeal lymph node;

* local nasopharyngeal or cervical enlarged lymph node was not considered as a distant lymph node.

### Acute adverse events of GC chemotherapy and CIK treatment

The adverse events associated with GC chemotherapy mainly consisted of allergic reactions, hematologic toxicity and gastrointestinal reactions, as has been previously reported [17]. There was no significant difference between the two groups.

After CIK infusion, 32 patients (28.6%) in the GC+CIK group presented with fever, diagnosed as a body temperature in the range of 37.5°C to 38.5°C. The fever did not last more than 6 h. Twelve patients developed mild chills that subsided after symptomatic treatment. No nausea, vomiting, rash, chest distress, anhelation, headache, or bellyache were found in these patients. After maintenance CIK treatment, no obvious functional damage was detected in major organs such as the heart, lung, liver and kidney. In addition, there was no acute or chronic infectious case in this study due to CIK infusion.

### Characteristics of CIK cells

After expansion, the final number of CIK cells produced was approximately 1×10^10^ to 1.5×10^10^. Cell viability was over 95%.The percentage of CD3^+^ T cells was over 80%, the percentage of CD3^+^CD8^+^ T cells was over 60%, and the percentage of CD3^+^CD56^+^ T cells was over 10%. No contamination by bacteria, fungi or endotoxin was detected.

### Survival analysis

#### Progression-free survival (PFS)

**Fig 3:**
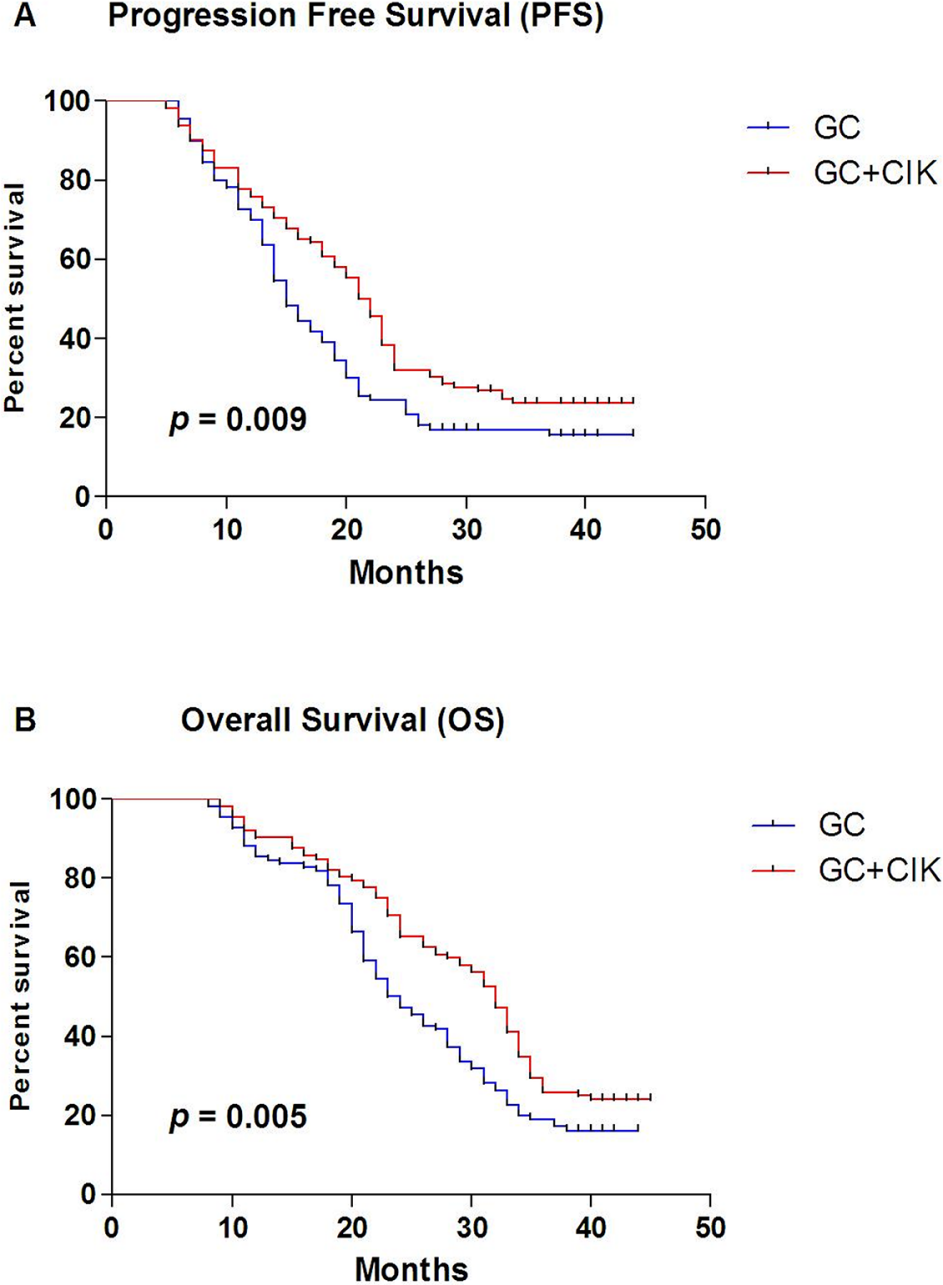
Kaplan-Meier survival curves during the 3-year follow-up period. Both progression free survival (PFS) (A) and overall survival (B) were better in Arm 1 (GC+CIK group, n = 112) than in Arm 2 (GC chemotherapy alone group, n = 110).

In the GC+CIK group, the 1-, 2- and 3-year PFS rates were 76.0%, 32.1% and 23.8.0%, respectively, while in the GC group these rates were 70.0%, 24.5% and 17.0%, respectively (Fig 3A). The median PFS was 21 months in the GC+CIK group and 15 months in the GC group. There was a significant difference between the two groups (log-rank test, p = 0.009), with the GC+CIK group showing a significantly improved PFS rate compared with the GC group.

#### Overall survival (OS)

The 1-, 2- and 3-year OS rates in the GC+CIK group were 90.2%, 65.2% and 25.9%, respectively, and these rates in the GC group were 85.5%, 47.3% and 19.1%, respectively **(Fig 3B)**. The median OS was 32 months in the GC+CIK group and 23 months in the GC group. There was a significant difference between the two groups (log-rank test, p = 0.006), with the GC+CIK group showing a significantly improved OS rate compared with the GC group.

## Discussion

As a pyrimidine analogue, GEM was mainly used in the clinical treatment of non-small cell lung cancer, breast cancer, and pancreatic cancer, among other cancers. It was only in the last decade that its short-term effects in the treatment of recurrent or metastatic nasopharyngeal carcinoma became known [21, 22]. However, the long-term effects are still not satisfactory. After GEM chemotherapy, 30% of NPC patients have been reported to develop progressive disease within 1 year, with the 3-year overall survival rate being less than 20% [4, 22]. Accordingly, there is a need to find more effective therapeutic strategies for recurrent or metastatic NPC, especially in terms of improving the long-term prognosis.

In our previous study, we found that GC chemotherapy combined with CIK transfusion can effectively improve the clinical outcome of post-radiotherapy distant metastasis of NPC patients compared with GC treatment alone.^17^ Because that study was limited by a small sample size, the therapeutic effects and long-term efficacy remained controversial. In the present study, through retrospective analysis of a relatively larger sample size of 222 patients, we evaluated the efficacy of CIK immunotherapy as a sequential treatment after GC chemotherapy for metastatic NPC patients after radiotherapy. Similar to our previous study [17], over the 3-year follow-up period, we found that the GC+CIK group had significantly higher survival rates (including PFS and OS) than the GC alone group, which indicated that CIK adjuvant immunotherapy could effectively maintain disease stability and prolong the survival of advanced NPC patients. Our results further confirmed the efficacy of CIK transfusion as adjuvant treatment to GC chemotherapy for metastatic NPC patients. From the survival analysis, we found an interesting phenomenon in which the PFS curves start to separate at month 12. However, the OS curves start to separate at month 18, indicating that not all patients will progress quickly when they are recurrent. One explanation for this phenomenon is that if patients are recurrent, they tend to immediately receive therapy that does not include GC chemotherapy and CIK therapy, such as another chemotherapy regimen, loco-radiation, or surgical resection. A subset of patients will respond to the therapy for a period of time. Thus, this subset will have longer OS despite suffering recurrence. Whether these alternative treatments are better than GC chemotherapy alone or in combination with CIK transfusion requires further investigation.

CIK immunotherapy can effectively enhance the therapeutic effect of GC chemotherapy through multiple mechanisms. First, when a sufficient number of CIK cells are present, they can directly kill potential or residual cancer cells, including tumor cells that are resistant to chemotherapeutic agents [23,24]. Second, infused CIK cells can improve the immunological status of NPC patients who have undergone chemotherapy via production of inflammatory cytokines such as IL-2, IL-6, and IFN-γ [25], and enhancement of the immunosurveillance capacity of the host to prevent disease progression. Third, previous studies have shown that a number of chemotherapeutic agents, including GEM, not only directly kill tumor cells but can also sensitize tumor cells in order to make them more susceptible to immune effector cells [26]. Thus, CIK immunotherapy and GC chemotherapy work in a complementary and cooperative manner, thereby effectively enhancing antitumor efficacy.

It is known that NPC is an Epstein-Barr virus (EBV)-associated malignancy. Thus, there have been several reports that utilized EBV-specific CTL transfusion for NPC refractory to conventional treatments [27, 28]. However, the long-term survival rate remains to be improved. Consistent with our findings, a recent phase II study by Chia WK et al [29] found that gemcitabine and carboplatin chemotherapy followed by EBV-specific CTL transfusion achieves better survival outcome in NPC patients with metastasis and/or local recurrence. Compared to the EBV-specific CTL, CIK cells are not antigen specific T cells. However, CIK cells have several advantages. First, they are easy to culture and produce. With a simple cytokine cocktail, approximately 10^10 cells can be obtained within 2 weeks from an initial culture of 10^7 cells. Second, these cells possess strong antitumor activity and target a broad spectrum of tumors without MHC restriction. Third, minimal toxicity and no graft-versus-host disease are found when using allogeneic CIK cells for infusion. Thus, CIK cells could be a reasonable choice of adoptive immunotherapy for cancer patients. Our present data together with previous studies strongly indicate that immuno-effector T cell transfer, including EBV-specific CTL or unspecific effector T cells, in combination with chemotherapy are more beneficial to advanced NPC patients than other options.

## Conclusions

Adjuvant autologous CIK cell maintenance treatment can effectively enhance the efficacy of GC chemotherapy to improve the prognosis of NPC patients with distant metastasis.
